# Association Between Workarounds and Medication Administration Errors in Bar Code-Assisted Medication Administration: Protocol of a Multicenter Study

**DOI:** 10.2196/resprot.7060

**Published:** 2017-04-28

**Authors:** Willem van der Veen, Patricia MLA van den Bemt, Maarten Bijlsma, Han J de Gier, Katja Taxis

**Affiliations:** ^1^ Faculty of Science and Engineering Unit PharmacoTherapy, -Epidemiology & -Economics University of Groningen Groningen Netherlands; ^2^ Erasmus Medical Center Department of Hospital Pharmacy Erasmus University Rotterdam Netherlands

**Keywords:** BCMA, bar code-assisted medication administration systems, workarounds, medication administration errors, bar coded medication administration, medication safety, hospitals

## Abstract

**Background:**

Information technology-based methods such as bar code-assisted medication administration (BCMA) systems have the potential to reduce medication administration errors (MAEs) in hospitalized patients. In practice, however, systems are often not used as intended, leading to workarounds. Workarounds may result in MAEs that may harm patients.

**Objective:**

The primary aim is to study the association of workarounds with MAEs in the BCMA process. Second, we will determine the frequency and type of workarounds and MAEs and explore the potential risk factors (determinants) for workarounds.

**Methods:**

This is a multicenter prospective study on internal medicine and surgical wards of 4 Dutch hospitals using BCMA systems to administer medication. We will include a total of 6000 individual drug administrations using direct observation to collect data.

**Results:**

The project was funded in 2014 and enrollment was completed at the end of 2016. Data analysis is under way and the first results are expected to be submitted for publication at the end of 2017.

**Conclusions:**

If an association between workarounds and MAEs is established, this information can be used to reduce the frequency of MAEs. Information on determinants of workarounds can aid in a focused approach to reduce workarounds and thus increase patient safety.

**Trial Registration:**

Netherlands Trial Register NTR4355; http://www.trialregister.nl/trialreg/admin/rctview.asp?TC=4355 (Archived by WebCite at http://www.webcitation.org/6pqTLxc6i).

## Introduction

Minimizing the risks of prescribing and medication administration is important to enhance patient safety in hospitals [1-6]. Many hospitals have implemented information technology-based systems such as computerized physician order entry (CPOE) systems to reduce prescribing errors [7-10]. Some have also implemented electronic bar code-assisted medication administration (BCMA) systems to reduce medication administration errors (MAEs) [11-18]. BCMA systems are designed to contribute to patient safety through scanning of the bar code on the medication package and the bar code on the patient’s identification wristband to guarantee the 5 “rights” in patient medication administration: right patient, right medication, right dose, right route, and the right time. However, in practice, BCMA systems are not always used as intended, and so-called workaround occurs [19-23]. Kobayashi et al [24] defined workarounds as “informal temporary practices for handling exceptions to normal workflow.” Investigating the use of CPOE systems in hospitals, Niazkhani et al [25] described 42 types of workarounds. Koppel et al [26] documented 15 types of workarounds in the BCMA process, including affixing patients’ identification bar codes to computer carts and carrying several patients’ prescanned medications on carts. That study documented 31 roots of these workarounds. Research on workarounds in the BCMA process focused on the qualitative description of the extent and type of workarounds in the BCMA process [27,28]. Little research has been done to quantify the frequency of workarounds in the BCMA process and investigate the impact of workarounds on patient safety, in particular, MAEs as a potential consequence of workarounds. Furthermore, little is known about the potential risk factors leading to workarounds. Therefore, we designed a study aimed at determining the association of workarounds with MAEs. Our secondary objectives are to determine the frequency and type of workarounds and the frequency and type of MAE, and to identify potential risk factors for workarounds.

## Methods

### Design

This study is a multicenter prospective observational study in adult patients who are admitted to a participating hospital in the Netherlands and who have their medication administered by BCMA systems.

The regional medical ethics committee (Regionale Medisch Ethische Commissie Zorgpartners Friesland) approved the study protocol. Study data are coded to guarantee the privacy of the participants.

### Setting

All included hospitals have implemented CPOE [10] and BCMA systems. They use a variety of software packages, both for the CPOE and for the BCMA systems. As a consequence, procedures for prescribing and medication administration differ between hospitals. Table 1 summarizes the main characteristics. Medication administration procedures within a hospital vary slightly between wards because of differences in patient groups or tasks (eg, in some hospitals, short stay surgical patients do not wear wristbands, but these are attached to the medication cart).

The included hospitals use bar code-labeled unit dose systems to distribute medication to inpatients. In the pharmacy departments, pharmacy technicians dispense bar coded medication for individual patients into trays labeled with the patient’s name and bar code. Trays are placed in medication carts in which they are then delivered to the wards once a day (or more frequently). Wards do not have ward-based medication stock (except for emergency medication). One of the selected hospitals uses so-called bedside assortment picking carts [29]. A cart contains all the medication commonly used on the ward. With this system, nurses select the medication for administration during the medication administration rounds.

In general, there are 4 scheduled medication administration rounds in the participating hospitals: 6-10 AM, 10-2 PM, 6-8 PM, and 8-10 PM. Medications are administered by 1 nurse. Nurse trainees are supervised by registered nurses. In the participating hospitals, there are approximately 10-20 inpatients admitted on a ward served by a registered nurse and a nurse trainee. A large ward is split into smaller units each serving 10-20 inpatients, each aided by a registered nurse and a nurse trainee.

During a drug administration round, nurses select the prescribed medication for each inpatient from the prefilled trays or from the bedside assortment picking carts. In addition to the cart, nurses also take along the computer on wheels or the workstation on wheels to access the CPOE system during the drug administration round.

Inpatients do not use their own (out-of-hospital prescribed) drugs.

### Participants

The study will enroll patients admitted to the internal medicine and surgical wards of 4 Dutch hospitals in which a BCMA system is used to administer medication. To be eligible to participate in this study, a participant must meet the following criteria: be a hospitalized patient and receive medication on those nursing wards that are participating in this study. We will exclude patients younger than 18 years.

### Outcome Measures

The primary outcome measure of the study is the proportion of medication administrations with 1 or more MAEs. For this outcome, we will study the association between the MAE and the occurrence of 1 or more workarounds.

The secondary outcomes are the frequency and type of workarounds, the frequency and type of MAEs in the BCMA process, and the association of potential risk factors with workarounds.

**Table 1:** Characteristics of the medication administration systems in the participating hospitals.

Item	Hospital 1	Hospital 2	Hospital 3	Hospital 4
Software system	RH Dharma	ViPharma	Klinicom	Pharma
System screen layout	Fixed layout	Fixed layout	Fixed layout	User-controlled screen layout
Administration system	Bedside assortment picking cart	Cart with prefilled patient-labeled trays	Cart with prefilled patient-labeled trays	Cart with prefilled patient-labeled trays
Log-in procedure for nurse	Once; automatic log-out after 15 minutes of inactivity	Once for 1 session	Once for 1 session	Once for 1 session
Log-out procedure for nurse	Manual; automatic log-out after 15 minutes of inactivity	Manual	Manual	Manual
Built-in additional check by nurse’s colleagues	Extra log-in for another nurse built in	Not possible	Extra log-in for another nurse built in	Not described in the instructions
Signal/alert system	Scanner beep and scanner warning light	Computer beep	Computer beep	Computer beep
Patient has no bar code	Not described in the instructions	Manual patient selection	Manual patient selection	Manual patient selection
Patient selection per administration round	Once, by selection of patient; automatically deselected after all medication for that round is administered	Twice, by selection and active deselection of patient after medication administration	Once, by selection of patient; automatic deselection after all medication for that round is administered	Once, by selection of patient; automatic deselection after all medication for that round is administered
Medication in the cart has no bar code	Robot-packed bar coded medication ordered from pharmacy	Manual drug selection	Manual drug selection	Nurse can overrule the system using her or his access code and manually select drug
More than 1 unit of the same drug for the same time prescribed	Scanned once, then the number of tablets is manually adjusted	Every drug unit is scanned	Scanned once, then the number of tablets is manually adjusted	Scanned once; a pop-up appears asking for the other tablets to be scanned
Patient away or sleeping	Prescribed medication is placed at the patients’ bedside, registered as given, and checked at 2:00 AM	Medication not given and not registered; noted in memo field	Medication not given and not registered; noted in memo field	Not described in the instructions
One-half or one-quarter of a tablet prescribed	Tablet scanned, plus code “half” or “quarter” scanned on computer	Not described in the instructions	Tablet scanned, plus noted by nurse in memo field on the screen	Not described in the instructions
Instructions on screen for nurse from pharmacy or prescriber	On-screen memo field included (medication data level)	On-screen memo field included (patient data level)	On-screen memo field included (medication data level)	On-screen memo field included (medication data level)

We will collect the following potential risk factors for workarounds using a structured data collection form (Multimedia Appendix 1): nurses’ characteristics (experienced, trained, or student nurse; nurses’ satisfaction with BCMA), workload characteristics (number of nurses on the ward, number of patients served by that ward, number of medicines per round per patient, number of medicines for all patients per round per ward), BCMA system characteristics (time after implementation of BCMA system on that ward, bar code on medication unit dose), medication characteristics (Anatomical Therapeutic Chemical Classification System [ATC] code of the medication, drug administration route), and general characteristics (hospital type, ward type, time of ward round, patient age and sex). We will ask the supervisor of the ward for data on the nurses’ education and experience. We will extract the number of patients on the ward, the medication and ATC code, and the number of drugs to administer to each individual patient during the specific administration rounds from the CPOE system. We will ask the supervising hospital pharmacist for the other risk factors.

### Data Collection

We will use disguised observation [30-34] to collect data. A total of 3 trained observers (undergraduate students, writing their master’s thesis) from the School of Pharmacy, University of Groningen and Utrecht University, the Netherlands, will observe the nurses while they give drugs to inpatients. To prevent nurses adjusting their behavior in the BCMA process while under observation, the observer will be introduced as being on the ward to monitor the performance of the medication distribution system on that ward. The observer will take part in several planned medication administration rounds on that ward and also observe unscheduled medication administrations. The observer will randomly pick a medication administration round with a minimum of 3 rounds every day and a weekly minimum of 18 rounds. During the different rounds, the observer will observe as many different nurses as possible. To prepare for the observation, the observer will study the standard operating procedures or the applicable drug administration procedures of the specific ward and the agreements on the BCMA process of that ward. In practice, the observer will accompany the nurse who administers the medication using the BCMA system and observe the administration of each dose of medication to the patient. The observer will record the nurses’ actions of giving drugs to the patients (according to the forms in Multimedia Appendix 1,Multimedia Appendix 2, and Multimedia Appendix 3). After each observed medication administration round, we will collect a (printed) computer output of the medication for that specific patient, day, and round from the hospital’s electronic patient records. Consequently, we will compare observation records with the prescribed medication on this computer output and with available standard operating procedures of the BCMA process for that specific ward, to identify workarounds and MAEs. We designed an Access database in which we will record the observation data and which we will link to each patient’s prescription and medication data.

If the observer becomes aware of a potentially serious error, the observer will intervene for ethical reasons, but the data will be included in the study.

### Training of the Observers

We will train our observers by having them study relevant literature on observational techniques [19,30,34-40], perform practical observations in a nonparticipating hospital under the supervision of the research team, and complete a written theoretical exam. The observers will have to pass the exam scoring 8 out of 10 points, having two chances to pass the exam. In case of a second failure, he or she will not be able to observe. Each observer will do pilot observations in a participating hospital, supervised by 1 of the researchers, for 1 week on the wards, to become familiar with the BCMA process. Pilot observations will be discussed with the research team. These observations are meant as a final training of the observer. Pilot data will be discarded.

### Definitions and Classification

**Figure 1:**
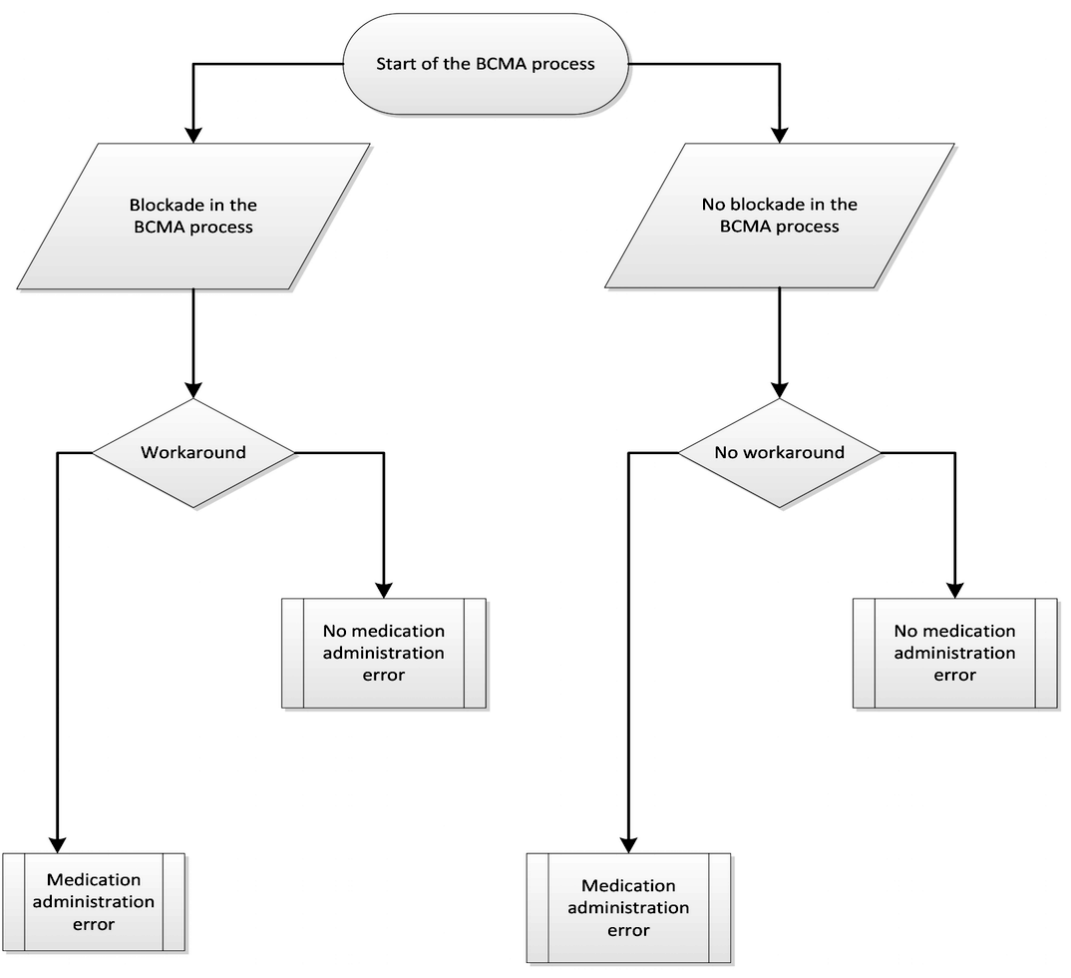
Flowchart of the bar code-assisted medication administration (BCMA) process in hospitals.

Workarounds are defined as “informal temporary practices for handling exceptions to normal workflow” for that specific ward and are operationalized as deviations from the available protocols [24]. Figure 1 depicts the BCMA workflow and the potential risk factors for workarounds in the BCMA process. We will classify workarounds using a self-developed classification system (Table 2) derived from the system of Koppel et al [26]. Workarounds can be related to patient identification, the scanning process, the alert signals, and other procedures, or can be work related. Allan and Barker [41] defined MAEs as “the administration of a dose of medication that deviates from the prescription as written (or ordered by CPOE) on the patient medication chart, or from standard hospital policy and procedures.” We will compare drug administrations with the doctor’s prescriptions as noted in the CPOE system in the pharmacy database. We will exclude intravenous and nonintravenous preparation errors because these errors are not preventable by BCMA and are thus unlikely to be influenced by workarounds in the BCMA process. We will classify the MAEs using the classification of van den Bemt et al [42] (Table 3). We will divide the number of erroneous medication administrations (containing 1 or more errors) by the number of observed drug administrations plus the number of omissions, thus using the concept of opportunities for errors as in other MAE research [43].

**Table 2:** Classification of workarounds in the bar code-assisted medication administration process^a^.

Workaround type	Example workaround
Procedure related: standard operating procedure, or procedure unclear or unknown	Nothing scanned
Patient related: no patient wristband or patient not in the room	Bed scanned, or loose wristband scanned, patient unscanned
Medication related: medication not bar coded	Unscanned, unidentified medication given
Nurse related: nurse disturbed	Nurse forgets patient or gives medication twice
Computer or scanner related: computer or scanner down or broken	Signals or alerts unseen, unscanned medication given
Other workarounds	Medication scanned for multiple patients; half tablets scanned as full dose

^a^Derived from Koppel et al [26].

**Table 3:** The most basic characterization of medication administration errors (MAEs)^a^.

MAE type	Example MAE
Omission	Drug prescribed, but not administered
Unordered drug administration	Drug administered, but not prescribed
Wrong dosage form	Drug dosage form administered to the patient deviating from prescribed dosage form: solution as an alternative to tablet
Wrong route of administration	Drug given by a wrong route of administration: oral liquid administered intravenously
Wrong administration technique	Drug administered using a wrong technique: intravenous push instead of intravenous infusion
Wrong dosage	Drug dosage too high or low: 20 mg instead of 20 μg
Wrong time of administration	Drug given at least 60 minutes too early or too late

^a^From van den Bemt et al [42].

### Sample Size Calculation

Prior studies [14,44-46] on the effect of BCMA show a substantial reduction (about 30%) of errors after the implementation of BCMA (from 14.4%, or 4743 errors in 32,972 observations, to 9.9%, or 2651 errors in 26,892 observations). The error rate of about 10% is a mix of all resulting errors, including those caused by workarounds. The purpose of our sample size calculation is to estimate the number of observations needed to reject the null hypothesis with a power of 90%. We performed a pilot study in 4 Dutch hospitals that were partially using BCMA (these hospitals did not participate in our final research) and found MAE rates, including time window errors caused by nurses and based on workarounds, fluctuating from 2% to 20% (2%, 4%, 5%, and 20%). We assume in our sample size calculation that 8% of medication administrations per patient per nurse result in a workaround. We also assume that the MAE rate associated with a workaround is 2-fold compared with the situation without a workaround; that gives us a relative risk of 2. With alpha of .05 and a power of 0.9, we need to observe 1500 individual medication administrations to patients per hospital to reject the null hypothesis.

### Data Monitoring

We will enter all data into an Access database (version 2010, Microsoft Corporation). The basis for the Access database will be the case report forms in Multimedia Appendix 1,Multimedia Appendix 2, and Multimedia Appendix 3. The first (Multimedia Appendix 1) is designed to collect data on potential risk factors for workarounds, the second (Multimedia Appendix 2) is designed to collect data on MAEs, and the third (Multimedia Appendix 3) is designed to collect data on observations of workarounds. These data will be made available to other researchers and editors on request. Data entry errors will be minimized by using multiple choice options and fixed data fields. At the end of the study, 10% of the entered data will be checked by a second researcher. If data entry errors are found, additional portions of 10% of the data will be checked until no errors are found within a portion. Also, a periodic backup of the study database of each hospital will be made and checked for missing data. Access to the research databases will be secured by passwords. Changing the format of the study documentation or study databases will be restricted to the primary investigator. New versions will be distributed from the central study location (the University of Groningen, the Netherlands). Before data analysis, we will lock the final database.

### Statistical Analysis

Data will be analyzed using IBM SPSS Statistics version 22 (IBM Corporation). We will analyze the potential association between workarounds and the occurrence of MAEs using univariate multilevel logistic regression, with the proportion of medication administrations with 1 or more errors as the dependent variable and the occurrence of workarounds as the independent variable. The nurse and the patient will be the levels in the multilevel analysis. We will analyze the occurrence of workarounds as a categorical variable, with the following categories: no workarounds (reference category), 1 workaround, 2 workarounds, and 3 or more workarounds. We will adjust for potential confounders by using multivariate multilevel logistic regression. The parameters in the multivariate multilevel logistic regression model will be hospital, ward type, day of the week, time schedule of medication administration rounds, ATC code, the number of drugs per patient per round, and the route of administration. We will report the adjusted odds ratio and 95% confidence interval. For the frequency and type of workarounds and MAEs, we will use descriptive statistics. Univariate and multivariate logistic regression will determine the association of the risk factors with the workarounds.

## Results

The project was funded in 2014 and enrollment was completed at the end of 2016. Data analysis is under way and the first results are expected to be submitted for publication at the end of 2017.

## Discussion

The Dutch BCMA study investigates the complex and multifaceted process of medication administration to hospital inpatients. Computer technology can assist not only the prescribing and dispensing of drugs, but also their administration. Several studies have shown that BCMA systems can contribute to patient safety in this final step of the medication distribution process [11-18]. On the other hand, computer technology can give rise to new MAEs, as is described in the literature [47]. Many of these errors occur at the human-machine interface, for example, due to inadequate training or understanding of the system or inadequate equipment. Such factors may lead to workarounds that may compromise patient safety. Although several articles have been published describing workarounds in a qualitative way, very little is known on whether they are associated with a higher risk of MAEs.

### Strengths and Limitations

The strength of the Dutch BCMA study is that it will provide quantitative information about workarounds and their possible association with MAEs, as one of the first studies worldwide, to our knowledge. Other strengths are the multicenter design, which enhances its generalizability, and the robust method of data collection by disguised observation.

There are some limitations and considerations, however. An important limitation, in general, is that the use of BCMA cannot prevent all MAEs. For example, BCMA systems will have no influence on the preparation of intravenous and nonintravenous medication. So, although this study will contribute to patient safety, further studies into other ways of preventing MAEs will remain necessary.

Although disguised observation is the best method for data collection in MAE studies, some limitations are associated with this technique. Despite thorough training of the observers, bias may still occur. To overcome observation bias, we considered the use of the work observation method by activity timing [34,48]. This elegant paperless method is used for time- and activity-based observations and is less suitable for observing workarounds and MAEs.

The observations may influence the nurse but, from the literature, we know that this effect (known as Hawthorne effect) [49,50] is small. The observer may also become tired and thus less accurate. How to train observers is not well documented in the literature. Patterson et al [19] performed an observational study in acute and long-term care wards using observers trained in ethnographic observations in complex settings. Other researchers trained nurse students as observers [51]. We will use all possible means, as well as the best possible literature base, to train the students.

We will try to reduce confounding by applying multivariate regression analyses (eg, hospital type, type of ward). However, in this type of observational study design, residual confounding may always remain [52].

Last but not least, we plan to conduct our research on internal medicine and surgical hospital wards. Although these nursing wards cover a broad range of patient categories, our findings cannot be generalized to all patient categories.

### Conclusion

BCMA has the potential to minimize the occurrence of MAEs, but workarounds may compromise this. Knowing how nurses overcome process barriers by using workarounds and their association with MAEs will produce opportunities to further increase patient safety in the process of BCMA.

## Appendix

Multimedia Appendix 1 Potential risk factors form.

Multimedia Appendix 2 Medication administration errors observation form.

Multimedia Appendix 3 Workarounds observation form.

## Abbreviations

ATC: Anatomical Therapeutic Chemical Classification System
BCMA: bar code-assisted medication administration
CPOE: computerized physician order entry
MAE: medication administration error
