# Supplementary material for: Association Between Workarounds and Medication Administration Errors in Bar Code-Assisted Medication Administration: Protocol of a Multicenter Study
Source: JMIR Res Protoc. 2017 Apr 28;6(4):e74. doi: 10.2196/resprot.7060 (PMC5429431; doi:10.2196/resprot.7060)
Supplement: Multimedia Appendix 1 [file resprot_v6i4e74_app1.pdf]

Appendix A: Potential risk factors form

Potential risk factors

Date and time: ……-……. - 20………   …………………….

Hospital: ……………………………………………………………………….

Ward: ……………………………………………………………………….

Patient number and gender:
……………………………………………………………………………M/F...

Date of birth: ……………………………………………………………………….

Nurse name: ………………………………………………………………………..

Drug name, form and strength: ….…………………………………………………….

……………………………………………………………………..………………

A nurse characteristics

- □ experienced nurse
- □ trained nurse
- □ student nurse
- □ nurse satisfaction with BCMA

B. workload characteristics

- □ number of nurses on ward ………………………………………………
- □ number of patients served by that ward …………………………………
- □ number of medications per round per patient ………………………….

- ☐ number of medication for all patients per round per ward ………………………..

C. BCMA characteristics

- ☐ time after implementation of BCMA on that ward ……………………..
- ☐ barcode on medication unit dose …………………………………..Y/N..

D. medication characteristics

- ☐ ATC code medication ……………………………………………………..
- ☐ drug administration route ………………………………………………

E. general characteristics

- ☐ hospital type ……………………………………………………………….
- ☐ ward type ……………………………………………………………………..
- ☐ time of medication administration round ……………………………….
