# Supplementary material for: Association Between Workarounds and Medication Administration Errors in Bar Code-Assisted Medication Administration: Protocol of a Multicenter Study
Source: JMIR Res Protoc. 2017 Apr 28;6(4):e74. doi: 10.2196/resprot.7060 (PMC5429431; doi:10.2196/resprot.7060)
Supplement: Multimedia Appendix 2 [file resprot_v6i4e74_app2.pdf]

Appendix B: Medication administration errors observation form

Medication errors observation form:

Date and time: ……-……. - 20………   …………………………………………

Hospital: ………………………………………………………………………….

Ward: …………………………………………………………………………. …..

Patient number and gender:
………………………………………………………………………….M/F ……..

Date of birth: ………………………………………………………………………….

Nurse name: …………………………………………………………………………..

Prescribed drug name, form and strength: ….
………………………………………………………………………………………..

………………………………………………………………………..………………

Dosage form given:

☐ Tablet
☐ Capsule
☐ Oral liquid
☐ Suppository/enema
☐ Injection
☐ Infusion
☐ Patch
☐ Ointment/cream etc.
☐ Eye/ear/nose drop

☐ Inhalation
☐ Other, namely…………………………………………………………………….

Strength of medication given: ……………………………………………………………………

Time-window:

☐ Time given ………………………………………
☐ Standard time (window 60 minutes before, 60minutes after the scheduled gift of medication)

Route of administration given:

☐ oral
☐ oral by gastric feeding tube
☐ rectal
☐ dermal
☐ local
☐ intravenous
☐ intramuscularly
☐ subcutaneous
☐ inhalation
☐ transdermal
☐ other, namely………………………………………………………………………

Number of tablets/capsules/suppositories given: ……………………………….

Injection-/infusion rate: ………………………………………………………………..
