# Supplementary material for: Association Between Workarounds and Medication Administration Errors in Bar Code-Assisted Medication Administration: Protocol of a Multicenter Study
Source: JMIR Res Protoc. 2017 Apr 28;6(4):e74. doi: 10.2196/resprot.7060 (PMC5429431; doi:10.2196/resprot.7060)
Supplement: Multimedia Appendix 3 [file resprot_v6i4e74_app3.pdf]

Appendix C: Workarounds observation form

Workarounds observation form:

Date and time: ……-……. - 20………   …………………….

Hospital: ……………………………………………………………………….

Ward: ……………………………………………………………………….

Patient number and gender:
……………………………………………………………………….M/F ……..

Date of birth: ……………………………………………………………………….

Nurse name: ………………………………………………………………………..

Drug name, form and strength: ….……………………………………………………

……………………………………………………………………..………………

Medication scanned:

- ☐ yes
- ☐ scanning barcode unit dose package separately from medication (medication already pealed out from the unit dose)
- ☐ no

Patient scanned:

- ☐ yes, in patient room
- ☐ yes, but not in patient room
- ☐ no

Scanning barcode attached to patient:

☐ yes

☐ yes but not scanable

☐ no

Overruling computer alerts or signals:

☐ yes

☐ no

☐ yes but alert or signal ignored

Alerts or signals not seen

☐ yes

☐ no

Confirming medication administration before the administration is given to the patient (not real-time):

☐ yes

☐ no

Scanning medication for more than one patient simultaneously:

☐ yes

☐ no

Intake of the medication left to the patient / medication in house:

☐ yes

☐ no

Hardware / software defect or deliberately disabled

☐ yes

☐ no
